# Virtual micro MLC commissioning

**DOI:** 10.1120/jacmp.v6i2.2032

**Published:** 2005-05-21

**Authors:** Laura Drever, Peter Dickof

**Affiliations:** ^1^ Medical Physics Department Allan Blair Cancer Centre Regina Saskatchewan Canada S4S 1V4

**Keywords:** multileaf collimator, leaf resolution, quality assurance, HD‐270

## Abstract

The resolution of multileaf collimators (MLCs) is limited by their finite leaf width. A commercial package (HD‐270™) uses 3D couch translation and leaf adjustments to emulate smaller leaf widths. In this paper, we report on the commissioning of this feature using software testing, dial gauge measurements, and film dosimetry. We also identify a variety of limitations: software bugs and truncation artifacts, MLC leaf positioning uncertainties (random variations, systematic gantry dependence and backlash), and uncertainties in couch positioning. These reduce the capabilities of this implementation below that achievable theoretically.

PACS numbers: 87.53.Kn, 87.53.Uv, 87.53.Xd

## I. INTRODUCTION

The finite leaf pitch of multileaf collimators (MLCs) limits their ability to conform to a blocked field shape.^(1)^ This has been identified as a major factor in the nonuse of MLCs on an MLC machine in the AAPM MLC survey.^(2)^ It has been proposed that conformation be improved by moving the patient perpendicular to the direction of leaf motion by less than the leaf spacing, then reconforming to the shape. This has been shown to be quite effective in both theory^(3)^ and measurement.^(4)^ The HD‐270™ is a complete clinical system developed for conveniently delivering such a treatment on Siemens Primus™ LINACs. This paper looks at the performance of the system and identifies some limitations in its use.

## II. METHODS

### A. HD‐270 configuration

In any modern treatment system, multiple hardware and software modules are involved, and there are potentially many combinations of such modules. The procedure we investigated for using the HD‐270 was as follows:

- The Pinnacle v6.2b planning system was used to produce treatment plans with multiple fields. In this system, a blocked outline was generated through either direct user input or automatic blocking of user‐identified and user‐entered critical structures. The MLC leaf shape for a single field was then conformed to the blocked outline. The HD‐270 capability of the LINAC was not explicitly modeled in this conformation process. The resulting MLC leaf shape is exported to a file in the Lantis™ RTP Link file format.
- The Lantis v5.22C2 Verify & Record system read the file produced by the planning system in the step above using the RTP Import module and loaded the setup parameters including the MLC leaf positions into the verify‐record database.
- The PRIMEVIEW™ module (v2.0.32) provided the interface between the Lantis database and the Primus treatment unit. The machine parameters required to produce an HD‐270 treatment were calculated using this software, and the Lantis database was updated accordingly. This software split each field into a sequence of either 2, 3, or 5 shifted subfields which delivered the same total monitor units (MU) but blurred the field edge by moving the couch 5 mm, 3 mm, and 2 mm, respectively. The parameters for these beams were then downloaded to the Primus treatment unit console in a convenient automatic manner.
- The Primus LINAC delivered the downloaded treatments after appropriate verification and sent the results back to the Lantis system for recording.
- The method of planning treatment and transferring data to the verify‐record system was thus no different from conventional treatment and will not be further discussed. This paper deals exclusively with the PRIMEVIEW software, which split a single field into an HD‐270 sequence of multiple subfields and with the Primus LINAC (including treatment couch) which delivered the sequence. Note that while intensity‐modulated radiotherapy (IMRT) and couch shifts can in principle be combined,^(5)^ the PRIMEVIEW software reviewed here does not support HD‐270 sequences during IMRT.
- In general terms, the HD‐270 sequence was created from a blocked field outline. In our configuration, no facility existed to import the outline from the planning system, and the outline could not be entered using a digitizer because the Lantis beam shaper module was not available to us. The PRIMEVIEW software caters for this; when the user wishes to generate an HD‐270 sequence, the software
- calculates the appropriate couch motions for the number of subfields indicated by the user,
- generates a blocked field outline based upon the MLC leaf positions that were transferred from the planning system, and
- calculates the new leaf positions required to match this blocked field outline at each of the couch position offsets.

In this paper, the correctness of these calculations and the accuracy of the system's components in making the required motions are assessed.

### B. The couch

The PRIMEVIEW software provides an effective MLC resolution *r*, allowing choices of 5 mm, 3 mm, or 2 mm. These distances give the displacement perpendicular to the direction of leaf and jaw motions required of the patient between the various subfields of an MLC sequence. This total translation may be composed of longitudinal, vertical, and lateral translations of the couch which will, in general, depend on the gantry angle $\theta_{g}$, the collimator angle $\theta_{c}$, the isocentric couch rotation $\theta_{i}$, and the eccentric, or pedestal, rotation of the couch $\theta_{e}$. The appropriate equations can be calculated using the matrix method of Siddon^(6)^ to be

(1) $$\begin{array}{rl} & r_{lat}\left(\theta_{g},\theta_{c},\theta_{i},\theta_{e}\right)=r\cos\left(\theta_{g}\right)\sin\left(\theta_{c}\right)\cos\left(\theta_{i}+\theta_{e}\right)\\ & \;\;\;+r\cos\left(\theta_{c}\right)\sin\left(-\theta_{i}-\theta_{e}\right) \end{array}$$

(2) $$\begin{array}{rl} & r_{lng}\left(\theta_{g},\theta_{c},\theta_{i},\theta_{e}\right)=r\cos\left(\theta_{g}\right)\sin\left(\theta_{c}\right)\cos\left(-\theta_{i}-\theta_{e}\right)\\ & \;\;\;+r\cos\left(\theta_{c}\right)\cos\left(-\theta_{i}+\theta_{e}\right),and \end{array}$$

(3) $$r_{vrt}\left(\theta_{g},\theta_{c},\theta_{i},\theta_{e}\right)=r\sin\left(\theta_{g}\right)\sin\left(\theta_{c}\right)$$

These equations reduce to those published previously^(7)^ when the isocentric and eccentric couch rotations are combined as a single angle. The HD‐270 takes both the isocentric and eccentric couch rotations into account. While these equations calculate the shifts required of the couch for an HD‐270 treatment, they cannot be delivered in practice because the Primus couch position is only known to the nearest millimeter; a rounding scheme must be established. This is not described in the PRIMEVIEW documentation. We tested the system at a variety of angles and inferred a rounding scheme from the results.

We used three dial gauges (resolution 0.001 in.=0.0254 mm) attached to a hydraulic lift placed on the floor plate of the couch to test the accuracy and reproducibility of the couch motion for 10 mm, 5 mm, 3 mm, 2 mm, and 1 mm shifts in the three axes. After the initial positioning of the couch, all subsequent motions were carried out using the automatic couch motion as programmed in the console. Dial gauge readings were recorded after each of 10 position shifts and the mean and standard deviations of the distances traveled were calculated. Previous studies of reproducibility of couch motion have tested motion to the nearest millimeter only.^(8)^

### C. The MLC

#### C.1 Creation of the blocked field outline

The HD‐270 software allows the creation of blocked field outlines using one of three different options called “Centered,” “Intrusive,” and “Non‐Intrusive.” The results of these operations were observed for a number of treatment fields in order to establish the exact algorithm used.

#### C.2 MLC shape creation

The MLC shape for each field segment is derived from the blocked field outline by moving leaf centers to intersect the outline in all cases. Leaf positions on the Primus MLC are specified in integer millimeters; a rounding scheme must be established. This is not described in the PRIMEVIEW documentation. We tested the system with a variety of offsets between neighboring leafs and effective resolutions and inferred a rounding scheme from the results.

#### C.3 The accuracy and reproducibility of leaf motion

The accuracy of the MLC leaf positioning in producing the subfields is determined by the reproducibility in positioning. “Picket fence” films were irradiated by multiple strip exposures with edges matched at a series of positions across the field. In our sequence, 10 strips were used for each leaf pair. The strips were numbered starting with strip 0 closest to jaw X1 and strip 8 closest to jaw X2. The nominal X1 leaf positions were then (10, 10, 7.5, 5, 2.5, 0, −2.5, −5.0, −7.5) cm, and the X2 leaf positions were (−10, −7.5, −5, −2.5, 0, 2.5, 5.0, 10.0, 10.0) cm. Strips 0 and 9 were thus fully closed and were irradiated with only 1 MU. Strips 1 through 8 had a nominal width of 2.5 cm and were irradiated with 5 MU. We numbered the position where strips *i* and i+1 match as match position *i*, 1⩽i⩽7. The films themselves were placed in a holder in the block tray with 1/4‐in. nominal buildup; this allowed measurement at a variety of gantry angles.

The irradiation order of the strips could be changed. When the numerical order was used, both strip edges at a single match point approached that point from the same direction, minimizing backlash at every match point. An alternate sequence with the strips irradiated in the order 9, 1, 2, 8, 7, 3, 4, 6, 5, 0 maximized the separation due to backlash at odd match positions and minimized it at even match positions.

Analysis of these films was done by fitting the peaks/troughs in the exposed film at each match position with a model of the match intensity. The model simulated the beam intensity at position *x* as a sum of two edges:

(4) $$Edge[x,pos,width]=1-\frac{1}{2}ERF\left[\frac{2K(x-pos)}{width}\right],$$

where ERF is the error function

(5) $$ERF[x]=\frac{2}{\pi }\int_{0}^{x}e^{-t^{2}}dt$$

**Figure 1:**
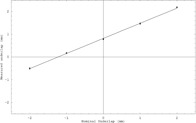
Calibration curve giving mean fitted beam underlap as a function of mean nominal underlap for picket fence films. Five films were exposed for nominal underlaps of 24 mm, 25 mm, and 26 mm and three films for 23 mm and 27 mm. The mean of the underlap over all leaf pairs and match positions is calculated for every film. The mean and standard deviation of the film means at each nominal underlap are plotted along with the fitted calibration curve. This curve shows a calibrated average beam underlap of −1.23 mm and a calibration factor of 1.50 (actual/measured).

“pos” is the position of the 50% intensity of the edge, and “width” is the width of the edge. The value of *K* was chosen to allow the specification of the width in millimeters from 10% to 90%, and the sign of *K* determined whether the edge intensity increased or decreased with increasing *x*. The fit for each leaf pair and position thus returned two widths and positions. Films were exposed with 0 mm, ±1 mm, ±2 mm nominal overlap, and the mean overall leaf pairs and match positions of the difference in fitted edge function positions (the shift) was then calculated for each film. The measured underlap was plotted against the mean observed shift to obtain a calibration curve as shown in Fig. 1. The fitted shifts in all films were multiplied by the inverse of the slope of this curve to obtain the reported under/overlap values.

### D. System performance

#### D.1 Dosimetric performance

We investigated the performance of the system using films of a 45° field edge generated by a stepped MLC, the angle most commonly used in the literature.^(3,^, ^4,^, ^7,^, ^9)^ The films were taken at the isocenter of a 6‐MV beam with 4 cm of solid water. When the collimator, gantry, and couch angles are all 0°, no round‐off is required; such cases were compared with those generated by an HD‐270 sequence with gantry, collimator, and couch angles of, respectively, 89°, 89°, and 359°. In that case, the algorithm truncated couch positions. Finally, these were compared with a sequence using couch positions set by hand to the rounded values. The films were digitized with a Vidar VXR‐12™ scanner, and the width of the effective penumbra from 20% to 80% was measured along with the amplitude of the oscillation of the 50% isodose curve.

#### D.2 Interaction with virtual wedge

Since the couch moves the patient during treatment, it is possible that the patient will be moved across the wedge direction for a wedged field changing the effective wedge factor. The virtual wedge factor was measured for a 10×10 cm field size, 100 MU treatment both without HD‐270 and with each HD‐270 resolution. While the same effect would occur with physical wedges oriented in the same direction, our physical wedges have their wedged direction perpendicular to the direction of patient motion; a small shift in the nonwedged direction has no significant effect on the wedge factor.

#### D.3 Treatment time comparisons

It takes longer to run HD‐270 treatments since the couch, the MLC, and the solid jaws all move between the individual fields of the treatment. Treatment times for a 100 MU, 10 MV, 10×10 cm treatment were measured using a stopwatch. The time from when the “Radiation On” button was pushed until the treatment completed was recorded and compared for a standard treatment field and for the same field divided into HD‐270 treatments.

## III. RESULTS AND DISCUSSION

### A. The couch

We have established that the rounding function is truncation and that the truncation is applied to the final position (i.e., the original position plus the calculated shift). The couch positions generated by HD‐270 will thus, on average, be displaced 0.5 mm from the ideal position in each of the three axes for a total average error of 0.9 mm. The maximum round‐off error will similarly be 1.0 mm in each direction for a total of 1.7 mm. For example, for $\theta_{g}=11.5^{{}^{\circ}},\theta_{c}=270.1^{{}^{\circ}},\theta_{i}=\theta_{e}=0^{{}^{\circ}}$, and the initial lateral position of ‐90 mm, longitudinal position of 730 mm, and vertical position of 10 mm, the calculated (lat, lng, vrt) displacements for a resolution of 5 mm are (−4.900 mm, −0.009 mm, 0.997) mm. After rounding, the actual shifts to the final positions were (−4, 1, 0) mm, for a total error of 1.67 mm. Measured couch shifts in the principal axes are compared with nominal values for a variety of small shifts in Table 1. The uncertainty in mechanical couch shift is ±0.3 mm.

**Table 1:** Mean and standard deviation of the measured travel of the Primus couch top

Programmed versus measured couch shifts (mm)					
programmed shift	10	5	3	2	1
lateral	10.10±0.07	5.24±0.10	3.02±0.16	1.83±0.10	0.76±0.23
longitudinal	9.77±0.07	4.68±0.13	2.82±0.05	1.78±0.04	0.73±0.14
vertical	9.99±0.08	5.00±0.13	2.98±0.06	1.99±0.07	0.76±0.19
combined RMS deviation	0.3±0.2	0.4±0.4	0.2±0.3	0.3±0.2	0.4±0.6

In the case where three subfields are chosen such that two have 3‐mm offsets from the central one, the spacing between groups of subfields is 4 mm. This means that the smoothing obtained will be intermediate between that obtainable for uniform 3‐mm and 4‐mm spacing. This is suboptimal, but no systematic errors are introduced.

## B. The MLC

### B.1 Creation of the blocked field outline

**Figure 2:**
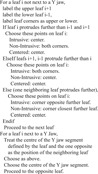
Pseudocode for generating blocked field shape from MLC leaf positions

**Figure 3:**
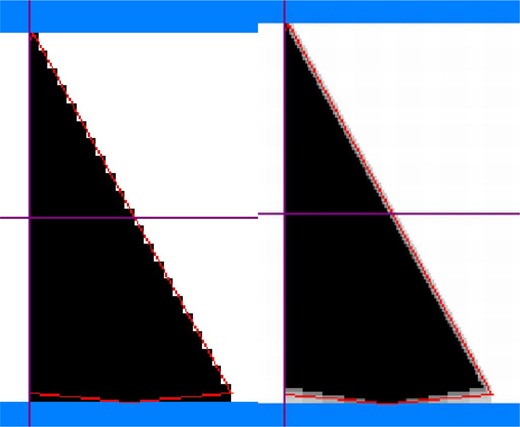
The original MLC leaf positions, the PRIMEVIEW‐generated blocked field outline and the resultant intensity map. Note that the choice of the midpoint of the field edge defined by the 7jaw at the bottom of the field results in a change in the field shape.

The pseudocode in Fig. 2 describes how the points of the blocked field outline are chosen. This algorithm is suitable for rounded shapes. As shown in Fig. 3, it will round off the straight edge of the field defined by the *Y* jaw unless the “Intrusive” option is chosen. This may not be clinically desirable. In particular, this will cause a problem if the edge in question is matched to one from another field (e.g., in a three‐field head and neck technique). It would be convenient to have options to either maintain the *Y* jaw edge unchanged by the production of the blocked field outline or to have the entire edge moved the same amount for each segment. The former would provide for a conventional match; the latter would provide an effectively broadened penumbra for the match.

#### B.2 MLC shape creation

We have established that the rounding function is truncation and that the truncation is applied to the final position (i.e., the original position plus the calculated shift). The leaf positions generated by HD‐270 will thus be 0.5 mm displaced from the ideal position on average. The MLC shape for each field segment is derived from the blocked field outline by moving leaf centers to intersect the outline in all cases. At this stage in the creation of an HD‐270 treatment there is an apparent bug. If any leaf should be at exactly +20 cm from the center, then that leaf and the opposing leaf will be positioned unpredictably. In one test case, a 10‐cm‐wide rectangular field was programmed so that one bank of leaves was positioned at the 20‐cm limit, and the other bank of leaves was positioned at 10 cm past the centerline of the collimator, creating a 40×10 cm field shifted 15 cm toward one leaf bank. The blocked field outline was derived properly for this MLC field, but the leaf positions calculated to best fit this outline produced a 40×10 cm rectangle shifted only 5 cm toward the leaf bank; each leaf bank was offset by 10 cm. In this case, the field completely missed the intended treatment volume. Since there was no shape change, it is possible that this would not be recognized if the setup was not viewed in the treatment room. In most cases, the shape changes that occur would be more easily recognized by the operator.

#### B.3 The accuracy and reproducibility of leaf motion

**Figure 4:**
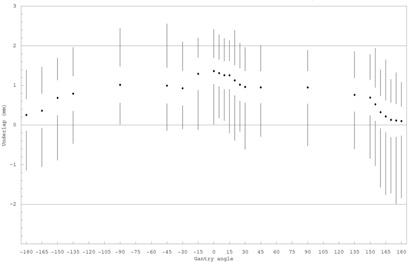
Mean under/overlap of picket fence fields measured at a variety of gantry angles. The data point represents the mean over all leaf pairs at all match positions, the clear space around each data point gives the standard deviation, and the vertical line shows the range of values. The horizontal line at ±2 mm gives the manufacturer's specified tolerance.

The mean of the variation in the under/overlap was measured ±0.4 mm, giving a mean error in leaf reproducibility of $\sqrt{0.4^{2}/2}=\pm 0.3\;\text{mm}$. A systematic variation in under/overlap was observed as a function of gantry angle, as shown in Fig. 4. A 1.3‐mm shift in the mean under/overlap was measured between gantry 0° to gantry 180° (IEC). Field sizes thus changed with gantry angle and were a minimum at 0° IEC. Most of the variation occurred within 30° of the 0 and 180 positions. It was observed that the change from 0° to $\pm 90^{{}^{\circ}}$ was concentrated in the leaf bank farthest from the floor. The shift has been reproduced on several occasions; the magnitude does not depend on the direction of rotation used to approach a given angle. The relatively large variation in leaf positioning accuracy revealed as a function of gantry angle does not appear to have been previously reported.

**Figure 5:**
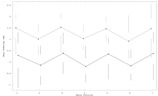
Mean under/overlap of picket fence fields measured at gantry angles of 0° (upper set) and 180° (lower set) at the positions where the eight‐strip exposures are matched. The sequence employed maximized the separation due to backlash at odd match positions and minimized it at even ones. The data point gives the mean over all leaf pairs at all match positions, the clear space around each data point gives the standard deviation, and the vertical line shows the range of values.

We investigated backlash as shown in Fig. 5. The mean difference in the positions of backlash maximum and minimum at a given gantry angle was measured to be 0.5 mm. Note that the separation between the gantry 0 and gantry 180 is roughly constant; the gantry variation is thus independent of this measured backlash.

### C. System performance

#### C.1 Dosimetric performance

**Figure 6:**
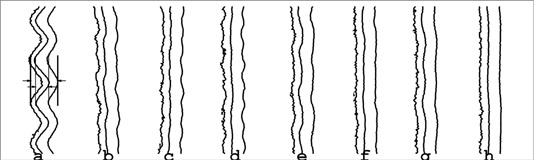
This the 80%, 50%, and 20% isodose lines and effective penumbrae of a 45° field edge generated by the MLC. All measurements were done using a source‐film distance of 100 cm and 4 cm solid water buildup. (a) no HD‐270 correction, 10‐mm resolution (effective penumbra 9.3 mm, 50% isodose oscillation 4.4 mm). (b) HD‐270, 5‐mm resolution, worst‐case truncation (effective penumbra 7.4 mm, 50% isodose oscillation 1.3 mm). (c) HD‐270, 5‐mm resolution, no round‐off (effective penumbra 7.4 mm, 50% isodose oscillation 0.6 mm). (d) HD‐270, 5‐mm resolution, worst‐case round‐off (effective penumbra 7.4 mm, 50% isodose oscillation 0.6 mm). (e) HD‐270, 3‐mm resolution, worst‐case truncation (effective penumbra 7.6 mm, 50% isodose oscillation 1.3 mm). (f) HD‐270, 3‐mm resolution, no round‐off (effective penumbra 7.2 mm, 50% isodose oscillation 0.6 mm). (g) HD‐270, 2‐mm resolution, worst‐case truncation (effective penumbra 7.4 mm, 50% isodose oscillation 1.3 mm). (h) HD‐270, 2‐mm resolution, no round‐off (effective penumbra 7.1 mm, 50% isodose oscillation 0.4 mm).

Figure 6 shows the 80%, 50%, and 20% isodose contours for several different combinations of angles and resolutions. The effective penumbrae for these fields range from 9.3 mm to 7.1 mm. For HD‐270 with 2‐mm and 3‐mm resolution, the introduction of the rounding scheme increased both the penumbra and the oscillation of the 50% isodose line. For HD‐270 with 5‐mm resolution, the 20% to 80% penumbra width was reduced from 9.3 mm to 7.6 mm regardless of which rounding scheme was used. The amplitude of oscillation of the 50% isodose contour, however, was measured to be 4.4 mm with no HD‐270 and only 1.3 mm when the HD‐270 with 5‐mm resolution was used at an angle where the truncation error was maximized. HD‐270 with 5‐mm resolution implemented with round‐off could achieve an oscillation as low as 0.6 mm when used at an angle where the round‐off error was maximized. This was not distinguishable in our test from the performance with no rounding. Note that some of the uncertainties identified above (e.g., the gantry angle variation) will affect all subfields equally. They will thus not affect the blurring of the field edge, resulting instead in a systematic shift in the blurred field edge which was not detected by this measurement.

#### C.2 Interaction with wedges

For resolutions of 2 mm and 3 mm, three‐ and five‐beam segments are used by the HD‐270 system, with an equal number of segments moved in each direction along the wedged direction of the virtual wedge. In such cases, the changes in effective wedge factor will tend to cancel. The central axis wedge factor for a 30° wedge was found to be 0.994±0.0007 in the 3‐mm resolution and 0.986±0.0012 in the 2‐mm resolution HD‐270 mode. The variation of 0.8% observed at 2 mm is somewhat larger than that observed in normal virtual wedge operation, but considered acceptable. For a resolution of 5 mm, only one segment is shifted, and the effective wedge factor change could be substantial. As a safety measure, the PRIMEVIEW software does not allow HD‐270 to be used in the 5‐mm mode with a wedge oriented perpendicular to the leaf motion.

The virtual wedge on the Primus requires a minimum number of monitor units to run. This limitation increases for small wedge angles. For a 15° wedge, the minimum numbers are 29 MU at 6 MV and 56 MU at 10 MV. For an HD‐270 treatment, these values will be doubled at 5‐mm resolution, tripled at 3‐mm resolution, and multiplied by 5 at 2‐mm resolution. This will make HD‐270 and virtual wedge mutually incompatible in some clinical cases.

#### C.3 Treatment time comparisons

On average, it took 23.4±0.2 s to deliver 100 MU at 10 MV when the field was not broken into HD‐270 treatments. For the same number of monitor units and the same energy, it took an average of 39.8±0.3 s to deliver an HD‐270 treatment with 5‐mm resolution. For the 3‐mm resolution, the average time was 50.5±0.6 s, and for the 2‐mm resolution the average time for the treatment was 76.2±0.4 s. A straight‐line fit of the times versus the number of couch motions required gave a mean time per couch motion of 12.9±0.6 s.

## IV. CONCLUSION

The HD‐270 is a complete clinical system for conveniently delivering a treatment with increased MLC conformation. To determine the quality assurance required to ensure that this system works correctly, the software that calculates the couch motion, derives the MLC contour, and matches the MLC to the derived contour was tested.

The algorithm that derives the MLC contour from the current leaf positions was tested and found suitable for producing rounded shapes. However, the algorithm will round off the straight edge of the field defined by the *Y* jaw unless the “Intrusive” option is chosen, which may not always be desirable. This will cause a problem if the edge in question is matched to another field. This step and its limitations could be avoided via the beam shaper module, but we do not have this module and were unable to test it.

Once the MLC contour is derived, the leaves are adjusted to best fit the derived contour. There appears to be a bug in the software that determines the required movements of the leaves. If any of the leaves are positioned at their outer extreme position (+20 cm), that leaf will close to some unpredictable location. This problem can be avoided by limiting the extreme position of the leaves to 19.9 cm when using the HD‐270 software.

It was found that the HD‐270 treatment introduces a small change to the virtual wedge factor for 2‐mm and 3‐mm resolutions and cannot be used with 5‐mm resolution. It takes longer to deliver the same number of monitor units when they are divided into HD‐270 segments than when the monitor units are all delivered to the initial field. Since the couch and MLC leafs can only move in whole millimeter increments, the algorithm that calculates the motions must implement a rounding scheme. Through testing it was determined that the rounding scheme used is truncation. The presence of truncation errors means that some of the benefit of HD‐270 is unnecessarily lost. In addition, our tests of the hardware components of the HD‐270 system showed uncertainties in positioning of the hardware. The individual uncertainties of ±0.9 mm couch position rounding, ±0.3 mm couch positioning error, ±0.3 mm MLC leaf reproducibility, ±1.2 mm MLC gantry variation, and ±0.25 mm mean MLC backlash add in quadrature to approximately ±1.7 mm. This is quite close to the 2‐mm resolution the system is designed to achieve. Worse, many of these uncertainties are systematic, depending on the particular beam orientation.

A clear understanding of the detailed operation of the entire software/hardware system is required in order to characterize the performance of such a system. In the case of HD‐270, there are significant determinants of system performance which can be missed by tests that do not select appropriate gantry, collimator, and floor angles.

Ongoing machine quality assurance for the use of HD270 requires little more than what is already required in national quality assurance documents for MLC and couch positioning. The remaining problems identified are limitations imposed by the software; these must be reviewed when software upgrades are performed. An annual review of the entire system performance via film exposure would also be worthwhile

The HD‐270 technique would perhaps be most useful in a conventional three‐field head and neck treatment where the shielding of the spinal cord is of significant clinical import. The opportunity to smooth the shielding and simultaneously “feather” the inferior edge of the lateral fields and the superior edge of the anterior field so as to minimize any mismatch is attractive.

However, in view of the limitations of the technique as implemented in our configuration and the work required to simulate the effect of such a treatment in the planning system, we have elected to not make clinical use of this option at this time. IMRT would appear to minimize the need for HD‐270 and be a more generally useful technique. Note, however, that the large variation in picket fence overlap as a function of gantry angle would appear to imply that, on a Primus platform, a) patient‐specific quality assurance on individual fields should be done at the prescribed gantry angle and b) a combined‐field film is necessary to assess the impact of any systematic shifts.
